# Analysis of related factors of portal vein thrombosis in liver cirrhosis

**DOI:** 10.1186/s12876-022-02632-z

**Published:** 2023-01-30

**Authors:** Xiaotong Xu, Jinglan Jin, Yuwei Liu, Hang Li

**Affiliations:** Department of Hepatology, The First Hospital of Jilin University, Changchun, China

**Keywords:** Liver cirrhosis, Portal vein thrombosis, Thromboelastography, IL-6, TNF-α

## Abstract

**Background and aims:**

To investigate the usefulness of interleukin-6 (IL-6), tumor necrosis factor-alpha (TNF-α), protein C (PC), and thromboelastography (TEG) to serve as a predictor of portal vein thrombosis (PVT) in patients with liver cirrhosis. Additionally, we examined the clinical significance of the above indicators in terms of disease progression.

**Methods:**

A total of 123 patients with liver cirrhosis were recruited from May 2021 to December 2021, according to the imaging findings. They were divided into the PVT group (n = 52) and the non-PVT group (n = 71). Furthermore, patients with PVT were divided into plasma transfusion groups (n = 13) and non-plasma transfusion groups (n = 39). The basic general information, past medical history, laboratory, and imaging examination data were collected and analyzed.

**Results:**

In univariate analysis, there was no significant difference between the two groups in IL-6, PC, reaction time (R), alpha angle (Angle), maximum amplitude, or coagulation index (CI) (P > 0.05). TNF-α in the PVT group was significantly lower than that in the non-PVT group (P = 0.001). K-time (K) in the PVT group was significantly higher than that in the non-PVT group (P = 0.031). There was no significant difference in IL-6, TNF-α, PC, or TEG between different Child–Pugh classification groups (P > 0.05). There were no significant differences in TEG between the plasma transfusion group and the non-plasma transfusion group. In Binary logistic regression analysis, TNF-α (OR = 0.9881, 95%CI = 0.971, 0.990, P < 0.001), K(OR = 1.28, 95% = 1.053, 1.569, P = 0.014), activate partial thromboplastin time (APTT) (OR = 0.753, 95%CI = 0.656, 0.865, P < 0.001), portal vein diameter (OR = 1.310, 95%CI = 1.108, 1.549, P = 0.002)and the history of splenectomy or embolism (OR = 7.565, 95%CI = 1.514, 37.799, P = 0.014)were related to the formation of PVT.

**Conclusions:**

TNF-α, K, APTT, portal vein diameter, and splenectomy or embolism history were associated with PVT formation, but IL-6 was not.

## Introduction

The term portal vein thrombosis (PVT) refers to the thrombosis of the main portal vein and/or the left and right branches of the portal vein, with or without mesenteric vein and splenic vein obstruction. PVT may accelerate the deterioration of liver function and increase the complications of portal hypertension [1].

In the current research, it has been proposed that PVT is primarily caused by a slowdown in blood flow velocity, an injury to local blood vessels, and a hypercoagulable state of the blood. PVT may also be associated with systemic inflammation and prethrombotic state (PTS). Thrombosis can induce inflammation to a certain extent, and inflammation may aggravate the hypercoagulable state of the blood [2]. It has been reported that IL-6 and TNF-α, familiar inflammatory factors, are higher in patients with PVT [3]. As a result, they may increase the risk of thrombotic diseases by enhancing platelet growth, promoting platelet adhesion, activation, and aggregation by activating neutrophils; activating endothelial cells; and affecting leukocyte adhesion and migration [4, 5].

A prethrombotic state is characterized by a coagulation and anticoagulation system disorder due to many factors and is also easy to become thrombosed [6]. Conventional coagulation tests (CCTs) fail to capture the full picture of coagulation because they do not account for anticoagulant components such as protein C (PC) and other cellular components including platelets. As a relatively new whole blood coagulation test, thromboelastography (TEG) includes reaction time (R), K-time (K), alpha angle (Angle), maximum amplitude (MA), and coagulation index (CI). Using these analysis techniques, the blood coagulation state [7] can be better evaluated by assessing coagulation kinetics (balance of procoagulant and anticoagulant factors), clot strength (platelet and fibrinogen,) and clot stability. Further research is needed to determine the clinical significance of TEG in patients with liver cirrhosis and portal vein thrombosis [8].

Currently, the diagnosis of PVT in cirrhosis patients is primarily based on imaging examinations, and serological diagnosis methods are lacking. As a result, the study on the factors affecting PVT will contribute to a better understanding of the disease, early detection of high-risk groups, and improved prognosis for patients.

## Method

### Patients

**Fig. 1:**
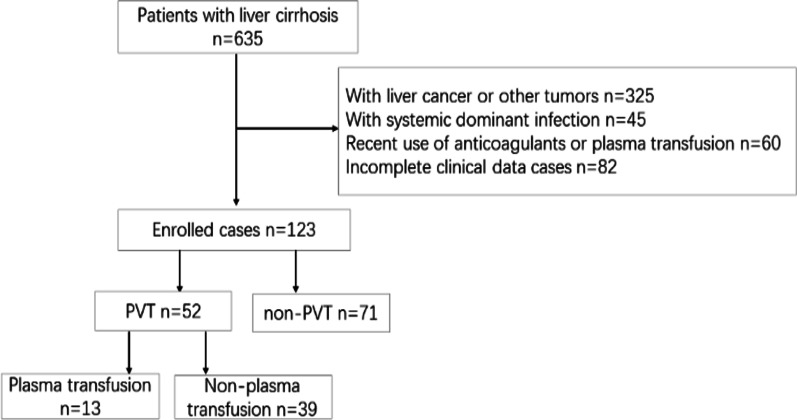
Flow diagram of the study population. PVT, portal vein thrombosis

We conducted a retrospective analysis of the patients admitted to the hospital for cirrhosis, both those with and those without PVT. From May 2021 to December 2021,123 patients (52 with PVT, 71 without PVT) were recruited, and their medical records were complete. Inclusion criteria: the diagnosis of liver cirrhosis [9] and PVT [10] must conform to the guidelines and consensus. Exclusion criteria: (1) patients without liver diseases complicated with PVT; (2) combined with liver cancer or other malignant tumors, blood system diseases; (3) after liver transplantation; (4) taking anticoagulant drugs or plasma transfusion recently (within 1 week); (5) along with dominant infection (Fig. 1).

### Laboratory tests

The peripheral veins were collected for blood specimens. Data collected from blood tests included the assessment of liver function, renal function, coagulation parameters, etiology of liver disease, IL-6, TNF-α, PC, TEG, ultrasound examination, and abdominal CT were performed on each patient. A model for end-stage liver disease (MELD) and Child–Pugh score was used to evaluate the severity of cirrhosis. The serum was stored and frozen at a temperature of − 80 °C. An enzyme-linked immunosorbent assay (ELISA) was performed using the Triturus ELISA analyzer. IL-6, TNF-α, and PC levels were determined using the manufacturer’s kit instructions with calibrators and samples processed (Human TNF-α ELISA kit, Human IL-6 kit, Human PC kit, Jingmei, Jiangsu, China).

Thromboelastography (TEG) is a whole blood test that analyzes the whole coagulation process and reports the function of plasma components, cell components, and coagulation factors. This instrument can analyze all hemostatic components, including coagulation kinetics, clot strength, and clot stability. It is more accurate in revealing the low and fragile "rebalance" state of blood coagulation in patients with liver cirrhosis. TEG was performed on whole blood using the TEG 5000 (Thrombelastograph Hemostasis Analyzer System, Haemonetics Corporation, Braintree, Massachusetts, USA) by experts blinded to patient information.

**Fig. 2:**
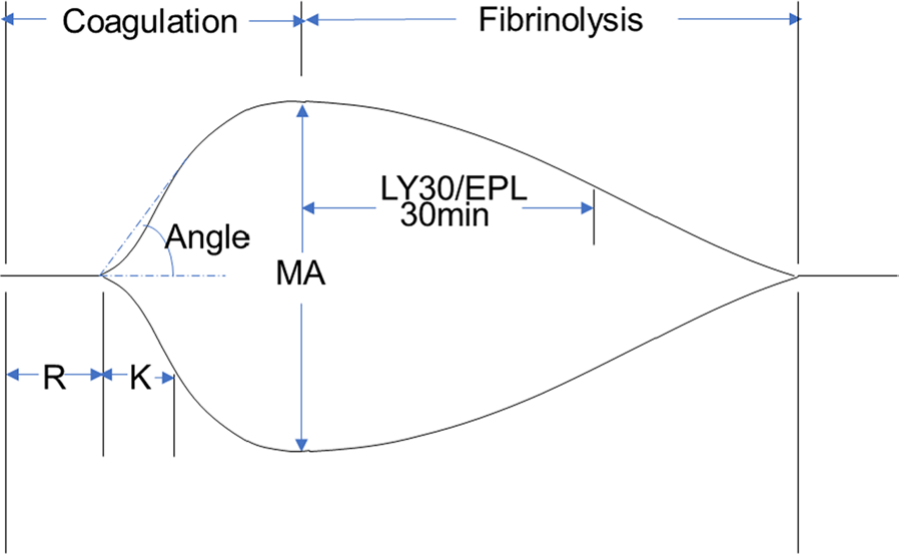
Thromboelastography (TEG). R, reactive time; K, K-time; Angle, Alpha angle; MA, Maximum amplitude; LY30, percent lysis 30 min after MA; EPL, Estimate Percent Lysis

A numbered of TEG variables, including clot time, clot formation time, alpha angle, maximum amplitude, and coagulation index, were examined by Haemonetics Corporation (Fig. 2). Reaction time (R) represents the rate of initial fibrin formation, which is linked to plasma clotting factor and circulating inhibitor activity. K-time (K) represents the clot kinetics. Alpha angle (Angle) is a measure of fibrin buildup and cross-linking speed and also represents fibrinogen concentration. K has an inverse relationship with Angle. Maximum amplitude (MA) refers to the maximum amplitude in a TEG trace. An extended R indicates that coagulation factors are deficient or that anticoagulants have been used. A shortened R indicates a hypercoagulable state. A prolonged K and a decreased Angle indicate a low level of coagulation. On the contrary, it indicates a state of high coagulation. LY30 and EPL represent fibrinolytic activity. The coagulation index (CI) represents the comprehensive state of blood coagulation, calculated by R, K, and MA.

### Statistics analysis

Univariate and Multivariate analyses were performed using SPSS.26.0 software. A binary logistic regression analysis was performed on the possible relevant indicators to summarize the relevant factors of PVT formation. The best diagnostic boundary value was identified by analyzing the ROC curve.

## Results

### Patient characteristics

There were no statistically significant differences between the PVT group and the non-PVT group regarding age, sex, etiological factors, diabetes, hypertension, spleen surgery history, ascites, Child–Pugh score, and grades (P > 0.05). There were significant differences in the patients' history of esophagogastric vein ligation or embolization, gastrointestinal bleeding, and hepatic encephalopathy (P < 0.05) (Table 1).

**Table 1:** Patient characteristics

	PVT (n = 52)	non-PVT (n = 71)	z/t/x^2^	P-value
Gender (male/female)	38/14	46/25	0.952	0.329
Age in years	56.98 ± 9.26	55.42 ± 9.07	0.334	0.739
Etiology			8.79	0.118
HBV	30 (57.7%)	36 (50.7%)		
HCV	2 (3.8%)	6 (8.5%)		
Others	20 (38.5%)	29 (40.8%)		
Diabetes mellitus	10 (19.2%)	11 (15.5%)	0.296	0586
Hypertension	6 (11.5%)	11 (15.5%)	0.394	0.530
Splenectomy/embolization	9 (17.3%)	6 (8.5%)	2.199	0.138
Esophagogastric vein ligation/embolization	19 (36.5%)	13 (18.3%)	5.182	0.023
Gastrointestinal bleeding			7.295	0.022
Previous	28 (53.8%)a	23 (32.4%)b		
Complicated with	3 (5.8%)a	2 (2.8%)a		
Without	21 (40.4%)a	46 (64.8%)b		
Complicated with HE	2 (3.8%)	11 (15.5%)	0.308	0.038
Complicated with ascites	41 (78.8%)	52 (73.2%)	0.512	0.474
Blood transfusion	13 (25%)	16 (22.5%)	0.101	0.750
Child–Pugh score	7 (6,8)	8 (6,9.5)	− 1.239	0.215
Child–Pugh grades			− 0.936	0.349
A	16 (30.8%)	20 (28.2%)		
B	28 (53.8%)	33 (46.5%)		
C	8 (15.4%)	18 (25.4%)		

*HBV*, Hepatitis B Virus; *HCV*, Hepatitis C Virus; *HE*, Hepatic encephalopathy

### Laboratory and examination data

ALT, AST, DBIL, APTT, TT, and TNF-α levels in the PVT group were significantly lower than those in the non-PVT group (P < 0.05). D-D, K, and the diameter of the portal vein in the PVT group were significantly higher than those in the non-PVT group (P < 0.05). There was no significant difference in IL-6, PC, R, MA, and CI between the two groups (P > 0.05) (Table 2).

**Table 2:** Comparison of laboratory data between PVT and non-PVT

	PVT (n = 52)	non-PVT (n = 71)	z/t	P-value
WBC (× 10^9^/L)	3.02 (2.17, 5.22)	3.58 (2.71, 5.11)	− 1.567	0.117
RBC (× 10^12^/L)	3.52 ± 0.72	3.54 ± 0.74	− 0.245	0.807
HGB(g/L)	105.50 (83.5,121)	110 (93,127.5)	− 1.267	0.205
PLT (× 10^9^/L)	62 (49.5, 98.5)	70 (53.5, 111.5)	− 1.262	0.207
ALT (U/L)	22 (15.1, 32.85)	26.9 (17.7, 46.45)	− 2.148	0.032
AST (U/L)	30.2 (24.8, 46.1)	42.6 (29.15, 72.55)	− 3.292	0.001
γ-GT (U/L)	37.25 (19.95, 64.15)	43.4 (25.7, 105.15)	− 1.487	0.137
ALP (U/L)	93.55 (78.4, 134.75)	100.5 (83.6, 141.25)	− 0.771	0.441
ALB (g/L)	32.37 ± 4.83	31.86 ± 7.42	0.429	0.668
TBIL (umol/L)	21.95 (14.65, 35.85)	27.9 (16.7, 45.1)	− 1.684	0.092
DBIL (umol/L)	5.25 (3.8, 9.8)	7.6 (5.1, 16)	− 2.309	0.021
Scr (mmol/L)	62.65 (49.9, 75.15)	61.7 (55.25, 71.15)	− 0.422	0.673
Na^+^ (mmol/L)	139.25 (136.3,142.35)	140.5 (137, 142.75)	− 1.045	0.296
PT (S)	14.63 ± 1.74	15.28 ± 2.55	− 1.574	0.118
APTT (S)	27.55 (25.3, 29.25)	29.5 (27.2, 32.85)	− 3.208	0.001
TT (S)	18.2 (17.1, 19.1)	18.8 (17.85, 19.75)	− 2.540	0.011
PTA (%)	70.06 ± 13.84	68.37 ± 17.99	0.566	0.572
INR	1.22 (1.14, 1.35)	1.29 (1.16, 1.4)	− 1.291	0.197
FBG (g/L)	1.87 (1.59, 2.73)	1.83 (1.59, 2.49)	− 0.468	0.639
D-D (ug/mL)	3.14 (1.58, 7.8)	1.89 (0.71, 3.92)	− 2.734	0.006
CRP (mg/L)	10.68 (2.45, 23.24)	4.92 (2.14, 15.38)	− 0.751	0.453
IL-6 (pg/mL)	122.85 (66.24, 136.15)	117.90 (61.71, 197.50)	− 0.607	0.544
TNF-α (pg/ml)	106.65 (47.78, 133.65)	137.50 (55.65, 207.60)	− 3.259	0.001
PC (ug/ml)	66.77 (32.57,78.76)	65.16 (54.66, 152.8)	− 1.582	0.114
R (min)	5.20 (4.40, 6.35)	5.50 (4.55, 6.15)	− 0.305	0.761
K (min)	3.57 (2.15, 5.60)	2.60 (2.00, 3.62)	− 2.156	0.031
MA (min)	44.55 (36.75, 57.25)	48.90 (39.80, 56.35)	− 1.037	0.300
CI	− 3.20 ( − 6.55, − 0.15)	− 1.90 ( − 3.95, − 0.20)	− 1.551	0.121
DPV (mm)	16.00 (14.00, 20.50)	15.00 (13.00, 16.00)	− 3.09	0.002

WBC, white blood cell count; RBC, red blood cell count; HGB, hemoglobin; PLT, platelet; ALT, alanine aminotransferase; AST, aspartate aminotransferase; γ-GT, Glutamyl transpeptidase; ALP, alkaline phosphatase; ALB, albumin; TBIL, total bilirubin; DBIL, direct bilirubin; Scr, serum creatinine; PT, prothrombin time; APTT, activated partial thromboplastin time; TT, thrombin tim; PTA, Prothrombin activity; INR, International normalized ratio; FBG, Fibrinogen; D-D, D-Dimer; CRP, C-reactive protein; PC, protein C; R, reactive time; K, K-time; MA, Maximum amplitude; CI, Coagulation index; DPV, the diameter of the portal vein

### Binary logistic and ROC curve analysis

Considering the collinearity and clinical significance, previous gastrointestinal bleeding history, APTT, D-D, TNF-α, IL-6, K, the diameter of the portal vein, and the history of splenectomy or embolism were analyzed by Binary logistic regression analysis (LR stepwise forward method). Finally, the factors related to the formation of PVT included TNF-α (OR = 0.981, 95%CI = 0.971, 0.990, P < 0.001), K (OR = 1.28, 95%CI = 1.053, 1.569, P = 0.014), APTT (OR = 0.753, 95%CI = 0.656, 0.865, P < 0.001), diameter of portal vein (OR = 1.310, 95%CI = 1.108, 1.549, P = 0.002)and the history of splenectomy or embolism (OR = 7.565, 95%CI = 1.514, 37.799, P = 0.014)(Table 3).

**Table 3:** Cut-off value of factors related to PVT formation and area under ROC curve

	β	P	OR	95%CI	Cut-off	SE	SP	AUC
DPV (mm)	0.270	0.002	1.310	(1.108,1.549)	17.35	0.404	0.901	0.663
APTT (s)	− 0.283	< 0.001	0.753	(0.656, 0.865)	29.41	0.808	0.507	0.670
TNFα (pg/mL)	− 0.020	< 0.001	0.981	(0.971, 0.990)	149.25	1.000	0.451	0.672
K(min)	0.23	0.251	1.285	(1.053, 1.569)	3.750	0.481	0.831	0.614

	β	P	OR	95%CI	AUC
Splenectomy or embolization	2.024	0.014	77.565	(1.514, 37.799)	0.544

	β	P	OR	95%CI	Cut-off	SE	SP	AUC
ALL	4.610	0.035	100.45		0.429	0.827	0.789	0.872

*DPV*, the diameter of the portal vein; *APTT*, activated partial thromboplastin time; *K*, K-time; *SE*, sensitivity; *SP*, specificity; *AUC*, area under the curve.

**Fig. 3:**
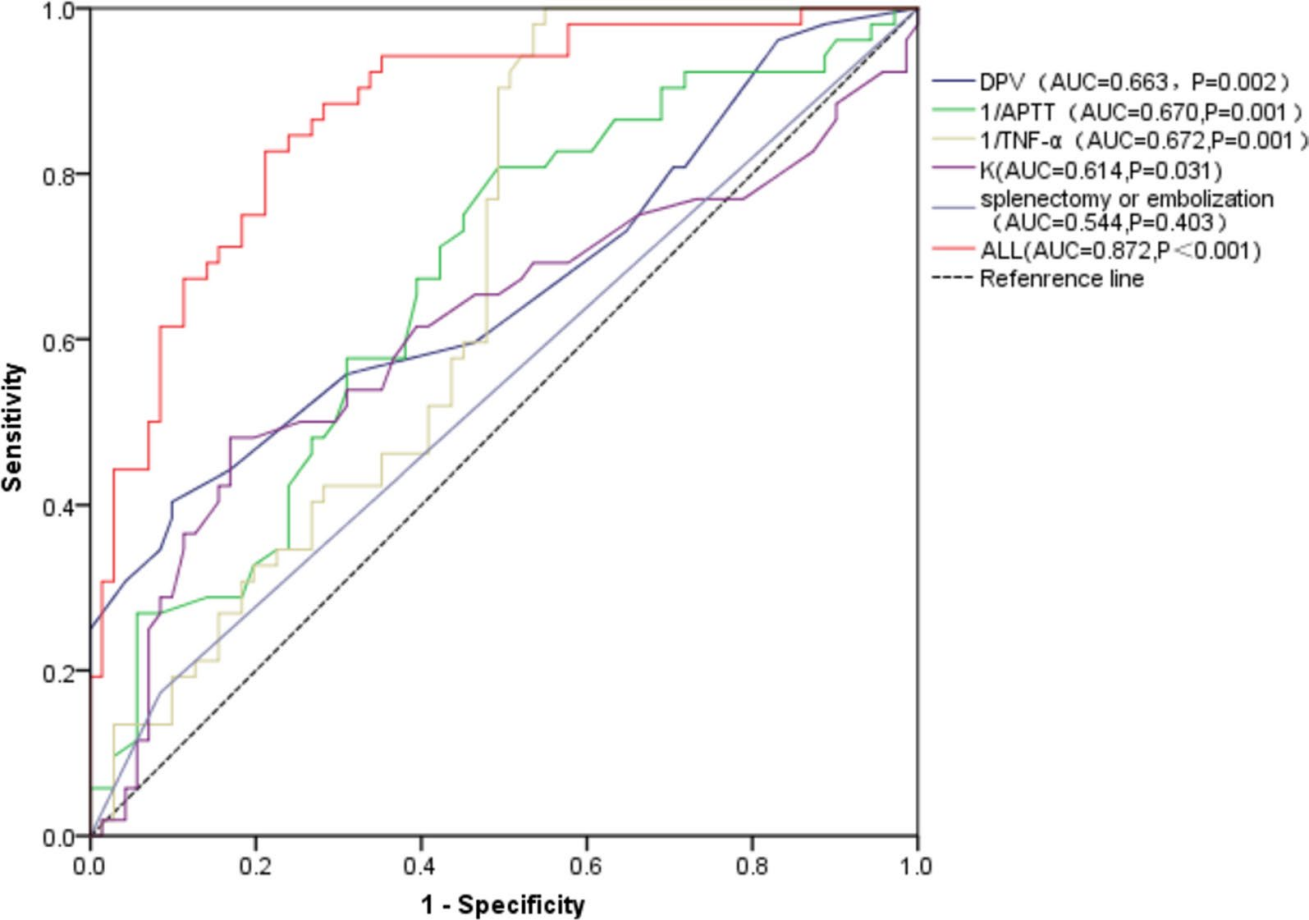
The area under the ROC curve of related factors. DPV, the diameter of the portal vein; APTT, activated partial thromboplastin time; PC, protein C; K, K-time

Then those related factors were analyzed by the ROC curve. The cutoff value of 1/TNF-α was 0.0067, with a sensitivity of 1 and a specificity of 0.451; K was 3.75 min, with a sensitivity of 0.481 and a specificity of 0.831; 1/APTT was 0.034, with a sensitivity of 0.808 and a specificity of 0.507; and the diameter of the portal vein was 17.35 mm, with a sensitivity of 0.404 and a specificity of 0.901, respectively. The risk of PVT was five times higher in patients with a history of splenectomy or embolization. Receiver operating characteristic curve analysis identified the AUC for TNF-α, K, APTT, the diameter of the portal vein, and history of splenectomy or embolization as 0.672, 0.614, 0.670, 0.663, and 0.544 respectively (Table 3) (Fig. 3). When they combined, the area under the ROC curve was 0.872 and the cut-off was 0.429 with a sensitivity of 0.827 and a specificity of 0.789.

### Comparison between Child–Pugh class A and Child–Pugh class B/C cirrhosis

In liver cirrhosis patients with PVT, there was no significant difference in IL-6, TNF-α, PC, and TEG among different Child–Pugh classification groups (P > 0.05) (Table 4).

**Table 4:** Relationship between IL-6, TNF-α, PC, and TEG and severity of liver disease

	Child-PughA (n = 16)	Child-PughB (n = 28)	Child-PughC (n = 8)	H	P-value
IL-6 (pg/mL)	124.4 (106.3,134.0)	124.6 (63.1,136.8)	89.29 (52.0,127.5)	1.425	0.490
TNF-α (pg/mL)	117.8 (48.0,136.4)	102.0 (48.1,132.7)	76.2 (47.4, 116.2)	1.339	0.512
PC (ug/mL)	66.8 (49.5,74.2)	65.2 (40.3,93.4)	40.7 (12.9,93.7)	0.277	0.871
R (min)	5.31 ± 1.25	5.59 ± 1.45	5.43 ± 1.78	0.639	0.727
K (min)	4.30 (1.95, 5.85)	3.57 (1.98, 4.80)	2.85 (2.15, 9.43)	0.190	0.909
Angle (deg)	55.54 ± 15.41	51.88 ± 13.41	52.84 ± 17.62	0.368	0.832
MA (mm)	41.85 (36.50, 62.40)	44.35 (37.23,57.95)	45.1 (29.9,47.1)	0.305	0.858
CI	− 2.97 ± 4.38	− 3.26 ± 3.94	− 4.00 ± 5.66	0.011	0.995

*PC*, protein C; *R*, reactive time; *K*, K-time; *Angle*, Alpha angle; *MA*, Maximum amplitude; *CI*, Coagulation index

### Comparison between plasma transfusion and without plasma transfusion events

According to whether plasma was transfused or not, patients with PVT in liver cirrhosis were divided into the plasma transfusion group (n = 13) and the non-plasma transfusion group (n = 39). There was no significant difference in the parameters of TEG between the plasma transfusion group and the nonplasma transfusion group (P > 0.05). But APTT, PT, and INR in the plasma transfusion group were higher than those in the non-plasma transfusion group (P < 0.05), and PTA in the plasma transfusion group was lower than that in the nontransfusion group (P < 0.05) (Table 5).

**Table 5:** Comparison of coagulation indexes between plasma transfusion group and non-plasma transfusion group

	Plasma transfusion (n = 13)	Non-plasma transfusion (n = 39)	t/z	P-value
R (min)	5.10(4.40, 7.20)	5.3 (4.45, 6.15)	− 0.148	0.882
K (min)	3.63(2.30, 5.90)	3.5 (1.85, 5.00)	− 0.645	0.519
Angle (deg)	49.23 ± 16.85	52.41 ± 13.69	0.684	0.497
MA (mm)	40.04 ± 14.14	48.51 ± 13.64	1.922	0.060
CI	− 4.65 ± 4.95	− 2.83 ± 4.01	1.330	0.189
TT(s)	27.00(24.70, 28.35)	18.40 (17.70, 18.80)	− 0.285	0.775
APTT (s)	29.20(27.60, 30.80)	27.00 (24.70, 28.35)	− 2.304	0.021
PT (s)	15.98 ± 1.53	14.18 ± 1.57	− 5.128	< 0.001
INR	1.35 ± 0.14	1.21 ± 0.13	− 3.576	0.001
PTA (%)	60.15 ± 10.54	73.36 ± 13.32	3.245	0.002

*R*, reactive time; *K*, K-time; *Angle*, Alpha angle; *MA*, Maximum amplitude; *CI*, Coagulation index; *TT*, thrombin time; *APTT*, activated partial thromboplastin time; *PT*, prothrombin time; *INR*, International normalized ratio; *PTA*, Prothrombin activity

### Correlation between traditional coagulation and TEG in PVT

**Fig. 4:**
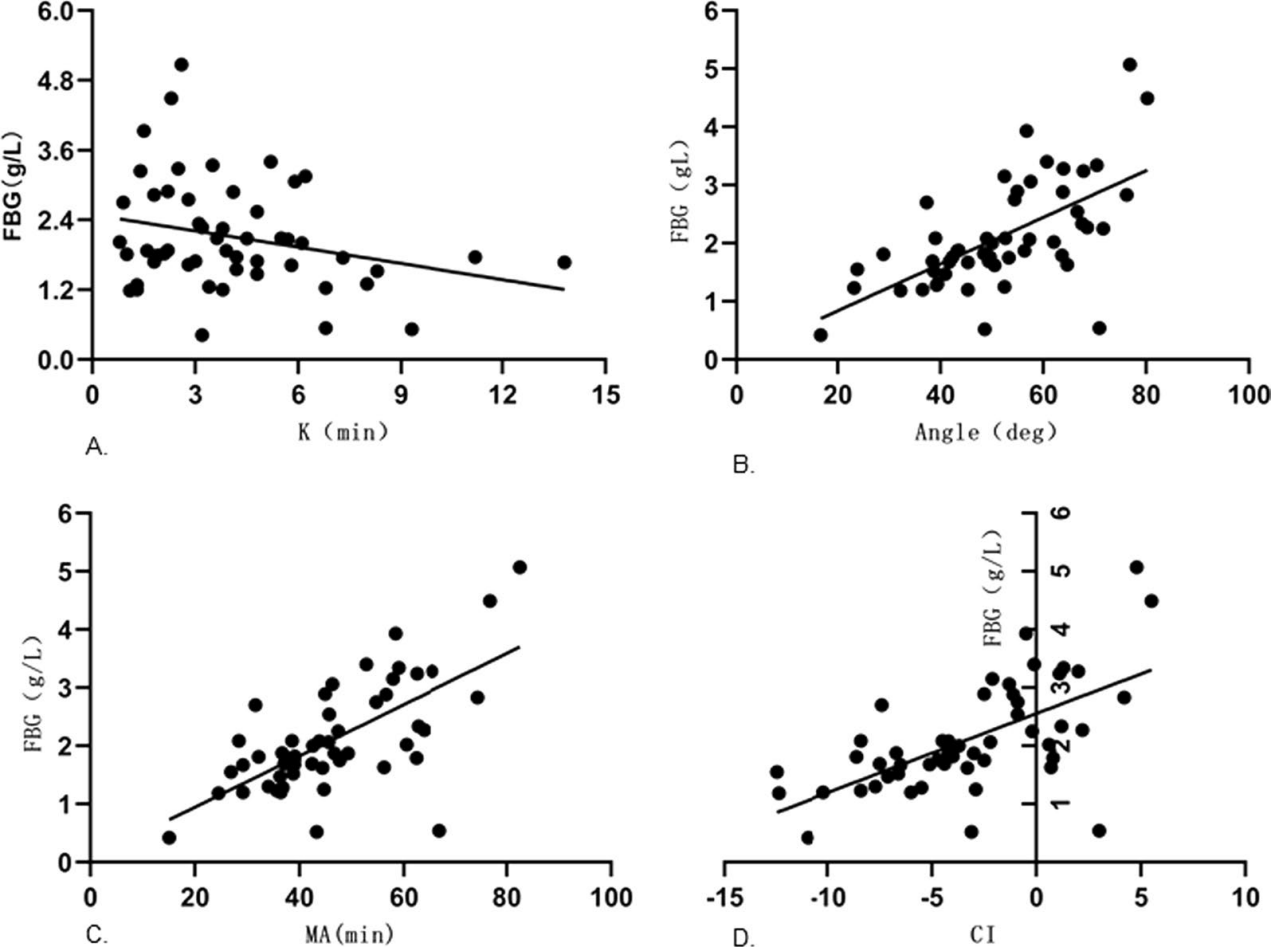
Scatter plot of significantly related indicators

In liver cirrhosis patients with PVT, FBG was significantly negatively correlated with K (r = -0.589, P < 0.05), significantly positively correlated with Angle (r = 0.639, P < 0.05), MA (r = 0.625, P < 0.05), CI (r = 0.632, P < 0.05) (Table 6) (Fig. 4).

**Table 6:** Correlation between traditional coagulation and TEG in patients with portal vein thrombosis

Variable/r	R(min)	K(min)	Angle(deg)	MA(mm)	CI
TT(s)	0.240	0.332^*^	− 0.370^*^	− 0.385^*^	− 0.377^*^
APTT(s)	0.402^*^	0.232	− 0.222	− 0.384^*^	− 0.349^*^
PT(s)	0.169	0.110	− 0.097	− 0.263	− 0.199
INR	0.236	0.166	− 0.159	− 0.314^*^	− 0.260
PTA (%)	0.237	− 0.164	0.158	0.310^*^	0.258
FBG(g/L)	− 0.456^*^	− 0.589^*^	0.639^*^	0.625^*^	0.632^*^

*stands for P < 0.05, and the difference is statistically significant. When |r|< 0.3, there is no correlation; 0.3 ≤|r|< 0.5 indicates low correlation; 0.5 ≤|r|< 0.8 indicates significant correlation|R|≥ 0.8 indicates high correlation

## Discussion

PVT is a common complication that can increase the bleeding rate, and deteriorate liver function. Currently, several studies are exploring the risk factors of PVT formation [11–14].When comparing the general data in our study, there were differences in the history of previous gastrointestinal hemorrhage and esophagogastric vein ligation or embolization between the two groups. However, multivariate analysis indicated that these factors were not independently associated with PVT**.** The formation of PVT after esophagogastric vein ligation or embolization may be related to the mechanical injury of the vascular endothelium [15] in one study**.**

Laboratory tests revealed that ALT, AST, and DBIL levels in the PVT group were significantly lower than those in the non-PVT group, but the above indexes were within the roughly normal range, contrary to the theory that liver function damage should be more serious when PVT occurs. There was no relevant literature report that addressed the relationship between transaminase and PVT. Considering that it may be related to the course of liver cirrhosis, the longer the course of liver cirrhosis, the lower the level of transaminase.

APTT, TT, and TNF-α in PVT were significantly lower than those in the group without PVT; the diameter of the portal vein, D-D, and K in the PVT group were significantly higher than those in the non-PVT group; the difference in PC and IL-6 between two groups were not statistically significant. As an anticoagulant, the synthesis of PC was reduced in patients with liver disease, but whether it was a risk factor for the formation of PVT remains controversial [16–18]. Even though the history of splenectomy or embolization was not statistically significant between the two groups, we included it in our multivariate analysis. PVT formation was associated with APTT, TNF-α, K, the diameter of the portal vein, and the history of splenectomy or embolization after multivariate analysis.

The decrease in APTT indicated that the blood was in a relatively hypercoagulable state. The diameter of the portal vein was related to its pressure. Blood flow is blocked and pressure is increased, when thrombosis occurs. As a result of compensating for the widening of the main portal vein, it will damage the endothelial cells and increase the risk of thrombosis. In most studies, it is believed that after splenectomy, the splenic vein becomes a blind end, which increases portal vein resistance, slows down the blood flow, and prolongs the contact time between coagulation factors and the blood vessel wall. The destruction and reduction of platelets after splenectomy led to a sharp rise in platelets [66]. Meanwhile, the operation itself will destroy the vascular endothelium, which together promotes the formation of thrombosis.

The relationship between PVT and inflammation has been controversial. It is unclear whether inflammation is the cause of venous thrombosis or the result of venous thrombosis [19]. IL-6 and TNF-α are familiar cytokines and some studies suggested that inflammation played a key role in the pathogenesis of venous thromboembolism [3, 20, 21]. The following three aspects [22] were considered to influence coagulation: down-regulation of physiological anticoagulant pathways, inhibition of fibrin removal, and activation of coagulation. The hemostatic balance would shift toward a prothrombotic state as a consequence. Besides, inflammation may increase the damage to endothelial cells.

But, interestingly, in our study only TNF-α was related to PVT formation, as a protective factor of PVT formation, different from previous research results [3, 14, 20]. On the one hand, it may be attributed to the small sample size, while on the other hand, it may be associated with different times of portal vein thrombosis. The time of thrombosis formation and the time between blood collection and the formation of PVT were difficult to determine, so the different states of PVT (acute or chronic) and the variable interval time (PVT formation to sample collection) may influence the level of TNF-α. TNF-α and IL-6 may both promote coagulation and anticoagulation [23, 24]. The study in mice found that IL-6 could stimulate macrophage proteolytic enzyme expression by activating the STAT3 pathway, thereby promoting thrombolysis [23]. TNF-α has also been found to have an antithrombotic effect in animal experiments [25]. Nosaka, M. found [24] that the TNF-α/TNF-Rp55 signal axis can regulate the dissolution of venous thrombosis. When TNF-α/TNF-Rp55 gene is deleted, thrombolysis will be inhibited. Therefore, we speculate that in our study, TNF-α had a dominant anticoagulant effect, whereas IL-6 had both procoagulant and anticoagulant effects, so further studies are necessary.

Hypercoagulability can be diagnosed based on at least two of the following four TEG parameters: shortened R, shortened K, increased Angle and increased MA [26]. According to the diagnostic criteria, our study suggested that PVT may not have a higher probability of hypercoagulability than non-PVT. On the contrary, the K of the PVT group was longer, and the overall coagulation level did not differ between the two groups, or the PVT group did not show significant hypercoagulability, as noted by Yanglan He [8].

In our study, we found that IL-6, TNF-α, PC, and TEG were not related to disease severity, the result of TEG was consistent with Yanglan He [8], but the results of IL-6 and TNF-α were different from Lee, F. Y. [27]. Comparing the coagulation indexes, APTT, PT, INR, and PTA in the plasma transfusion group were significantly higher than those in the non-plasma transfusion group (P < 0.05). But there was no significant difference in TEG between the groups (P > 0.05). We may be able to reduce blood transfusion and save blood products [28] if we considered the conventional coagulation test level and bleeding risk of patients before plasma.

However, our study has the following limitations. Firstly, this was a retrospective study in which a limited number of patients were included. Secondly, the accuracy of screening indicators cannot be validated in the other group. Finally, as the time of thrombosis was difficult to determine, it was uncertain whether it had an effect on observation indexes. Therefore, further research is needed.

## Conclusion

In our study, we found that TNF-α, APTT, K, the diameter of the portal vein, and the history of splenectomy or embolization were related to PVT, which can help early identify the population in the liver cirrhosis with a high risk of PVT.

## Data Availability

All data are available upon request.

## Abbreviations

IL-6: Interleukin-6
TNF-α: Tumor necrosis factor-alpha
PC: Protein C
TEG: Thromboelastography
PVT: Portal vein thrombosis
R: Reaction time
Angle: Alpha angle
MA: Maximum amplitude
CI: Coagulation index
K: K-time
PTS: Prethrombotic state
CCTs: Conventional coagulation tests
ELISA: Enzyme-linked immunosorbent assay
MELD: Model for end-stage liver disease
LY30: Percent lysis 30 min after MA
EPL: Estimate percent lysis
HBV: Hepatitis B virus
HCV: Hepatitis C virus
HE: Hepatic encephalopathy
WBC: White blood cell count
RBC: Red blood cell count
HGB: Hemoglobin
PLT: Platelet
ALT: Alanine aminotransferase
AST: Aspartate aminotransferase
γ-GT: Glutamyl transpeptidase
ALP: Alkaline phosphatase
ALB: Albumin
TBIL: Total bilirubin
DBIL: Direct bilirubin
Scr: Serum creatinine
PT: Prothrombin time
APTT: Activated partial thromboplastin time
PTA: Prothrombin activity
INR: International normalized ratio
FBG: Fibrinogen
D-D: D-Dimer
CRP: C-reactive protein
DPV: Diameter of the portal vein
SE: Sensitivity
SP: Specificity
AUC: Area under the curve
